# Replication independent DNA double-strand break retention may prevent genomic instability

**DOI:** 10.1186/1476-4598-9-70

**Published:** 2010-03-31

**Authors:** Narisorn Kongruttanachok, Chutipa Phuangphairoj, Araya Thongnak, Wanpen Ponyeam, Prakasit Rattanatanyong, Wichai Pornthanakasem, Apiwat Mutirangura

**Affiliations:** 1 Center of Excellence in Molecular Genetics of Cancer and Human Diseases, Department of Anatomy, Faculty of Medicine, Chulalongkorn University, Bangkok 10330, Thailand

## Abstract

**Background:**

Global hypomethylation and genomic instability are cardinal features of cancers. Recently, we established a method for the detection of DNA methylation levels at sites close to endogenous DNA double strand breaks (EDSBs), and found that those sites have a higher level of methylation than the rest of the genome. Interestingly, the most significant differences between EDSBs and genomes were observed when cells were cultured in the absence of serum. DNA methylation levels on each genomic location are different. Therefore, there are more replication-independent EDSBs (RIND-EDSBs) located in methylated genomic regions. Moreover, methylated and unmethylated RIND-EDSBs are differentially processed. Euchromatins respond rapidly to DSBs induced by irradiation with the phosphorylation of H2AX, γ-H2AX, and these initiate the DSB repair process. During G0, most DSBs are repaired by non-homologous end-joining repair (NHEJ), mediated by at least two distinct pathways; the Ku-mediated and the ataxia telangiectasia-mutated (ATM)-mediated. The ATM-mediated pathway is more precise. Here we explored how cells process methylated RIND-EDSBs and if RIND-EDSBs play a role in global hypomethylation-induced genomic instability.

**Results:**

We observed a significant number of methylated RIND-EDSBs that are retained within deacetylated chromatin and free from an immediate cellular response to DSBs, the γ-H2AX. When cells were treated with tricostatin A (TSA) and the histones became hyperacetylated, the amount of γ-H2AX-bound DNA increased and the retained RIND-EDSBs were rapidly repaired. When NHEJ was simultaneously inhibited in TSA-treated cells, more EDSBs were detected. Without TSA, a sporadic increase in unmethylated RIND-EDSBs could be observed when Ku-mediated NHEJ was inhibited. Finally, a remarkable increase in RIND-EDSB methylation levels was observed when cells were depleted of ATM, but not of Ku86 and RAD51.

**Conclusions:**

Methylated RIND-EDSBs are retained in non-acetylated heterochromatin because there is a prolonged time lag between RIND-EDSB production and repair. The rapid cellular responses to DSBs may be blocked by compact heterochromatin structure which then allows these breaks to be repaired by a more precise ATM-dependent pathway. In contrast, Ku-mediated NHEJ can repair euchromatin-associated EDSBs. Consequently, spontaneous mutations in hypomethylated genome are produced at faster rates because unmethylated EDSBs are unable to avoid the more error-prone NHEJ mechanisms.

## Background

We recently explored whether endogenous DNA double-strand breaks (EDSBs) are associated with genomic hypomethylation and genomic instability [1]. Complete or partial methylation of CpG dinucleotides in the human genome commonly occurs at interspersed repetitive sequences [2]. In cancer, interspersed repetitive sequence methylation is often reduced [2-7]. Spontaneous mutations, including loss of heterozygosity, chromosome translocation and DNA deletion, are associated with global hypomethylation in cancer. This genomic instability is also observed as a result of chemically- and genetically-induced demethylation processes [8-18]. Interestingly, these DNA lesions, which are the product of recombination between different loci, are mediated by DNA double strand breaks (DSBs).

Low levels of DSBs can occur spontaneously; these spontaneous breaks are known as endogenous DSBs (EDSBs) [1,19]. There are several possible mechanisms that produce EDSBs. γ-H2AX, the serine 139-phosphorylated form of histone H2AX, is one of the earliest DSB repair responses present on histone tails [20,21]. Several factors can influence the production of γ-H2AX foci, including a replicative DNA polymerase encountering single-stranded DNA breaks resulting in EDSBs, temperature, osmolarity, oxidative DNA damage, endonucleases [19,22-29], down-regulation of genes involved in DNA binding, ion flux, gene regulation and RNA processing [30].

EDSBs are usually considered hazardous to cells. However, there are some EDSBs that benefit cells. In 2003, Vilenchik and Knudson proposed that there are 5-10 EDSBs per cells [19]. However, the small number of EDSBs could play a key role in genomic instability in cancer, as these breaks can be intermediates in spontaneous genomic or chromosomal rearrangements in cancer [19]. Hazardous chemical agents and ionizing radiation produce large numbers of DSBs, which can be observed as fragmented DNA [31,32]. This breakage can trigger apoptosis, and errors in repair lead to mutations [33]. DSBs, however, do not play a role in heat- or hypertonicity-induced cell death [26,34]. In contrast, some EDSBs are derived from physiologic processes. V(D)J recombination is important in lymphocyte development [35], and topoisomerase II helps maintain genomic integrity [36].

**Figure 1:**
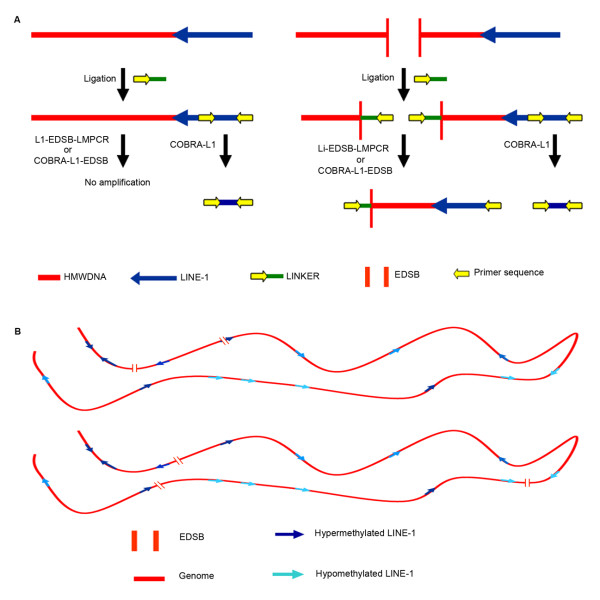
**Schematic representation of (A) EDSB-PCR and (B) L1-EDSB methylation status**. (A) Red lines, blue arrows and parallel vertical bars represent genomic DNAs, LINE-1 sequences and EDSB ends, respectively. First, LMPCR linkers, yellow arrow and green line, are ligated to EDSB ends. Yellow arrows are primer sequences. On the left, there is no EDSB and only COBRA-L1 yields a positive amplicon. On the right, only an EDSB end located nearby LINE-1 sequence is detected as L1-EDSB-LMPCR or COBRA-L1-EDSB (1). (B) The two red lines represent the same chromosomes of two different cells. Methylation levels of the LINE-1s are distinct among loci, but methylation levels between nearby LINE-1s are closely correlated (2). Blue arrows represent LINE-1 sequences, in which methylation levels of the dark blue LINE-1s are higher. Two parallel vertical lines represent EDSB ends. Detectable EDSBs can only be found rarely (from EDSB PCR data) and randomly (from variable EDSB PCR amplicon sizes (data not shown)); however, they are found preferentially near hypermethylated LINE-1s (1).

Recently, we developed a novel PCR technique to measure the number of EDSBs [1] by combining ligation-mediated polymerase chain reaction (LMPCR) [35] and intersperse repetitive sequence (IRS) polymerase chain reaction [37]. LMPCR is a technique designed for the analysis of locus-specific EDSBs during lymphoid development, such as V(D)J recombination [16,18,19] and hypermutation [20]. Without additional DNA restriction, double stranded DNA oligonucleotides linkers are ligated to the genomic DNA at existing EDSB ends. Then, EDSBs can be analyzed by PCR using primers located in the linker and in specific locus upstream/downstream of the EDSBs. In our technique, we substitute the locus specific primer with a primer located in IRSs in the PCR step (Fig 1A). Therefore, we could exploit the interspersed nature and the large number of IRSs in the human genome to measure the minute numbers of randomly distributed EDSBs (Fig. 1B). The EDSB PCR measures DSBs differently from the comet assay [31,32], pulse field gel electrophoresis [38] and γ-H2AX foci analysis [20,21]. While the detection of γ-H2AX foci, the formation of which represents one of the cellular responses to DSBs, the comet assay, pulse field gel electrophoresis and EDSB PCR measure the quantity of DSBs. High-dose radiation can produce positive results in comet assays and pulse field gel electrophoresis, as multiple small DNA fragments migrate away from the bulk of the genomic DNA. However, comet assay and pulse field gel electrophoresis cannot detect small numbers of randomly spaced DSBs because the DNA fragment size remains large and the majority of the chromosomes are intact.

A summary of results describing EDSBs detected by EDSB PCR [1] is provided in figure 1B and in additional file 1. EDSB PCR can be employed to identify and quantify the minute number of randomly distributed EDSBs. We identified EDSBs in all normal and cancer cells that we analyzed and in all cell phases. The majority of EDSB ends, blunt-ended and 5' phosphorylated [1], were similar to the signal ends that occur during V(D)J recombination [35] and hypermutation [39]. We chose to evaluate a subclass of interspersed repetitive sequences called long interspersed element-1 (L1 or LINE-1) sequences because the methylation status of these retrotransposable elements has been extensively studied [2,4,40]. The number and methylation state of EDSBs were analyzed for LINE-1 sequences near EDSBs in the L1-EDSB templates [1]. The L1-EDSBs of almost all tested normal and cancer cells were hypermethylated, meaning LINE-1s at sites closest to the EDSBs were more highly methylated than those at other sites in the genome [1] (Additional file 2). The DNA methylation preexists in the genome and may not be produced by the DNA breaks [1]. Moreover, although EDSBs were hypermethylated in most examined cell phases, hypermethylation was most significant during the G0 phase [1] (Additional file 2). This indicates that there exist EDSBs in non-replicating cells (replication-independent EDSBs; RIND-EDSBs), and that methylated and unmethylated forms of EDSBs may be processed differently. LINE-1 methylation levels are different among loci [2]. Consequently, L1-EDSB hypermethylation indicates that RIND-EDSBs are preferentially localized in methylated genomic regions (Fig. 1B). In contrast, EDSBs during S phase localize within less methylated genomic regions than in G0 [1]. DNA replication produces EDSBs from abnormal DNA lesions that can lead to mutations associated with cell transformation and cancer [19]. Therefore, the unexplored ramifications and processing of methylation related RIND-EDSBs warrant detailed investigation.

DSBs are processed by a number of DNA repair pathways, the choice of which depends partly on the phases of the cell cycle. Homologous recombination repair is precise, requires sister chromatids and is processed during DNA replication and in G2 phase [41]. Non-homologous end-joining (NHEJ) is thought to repair the majority of DSBs and uses fast, but error-prone, re-ligation of the two broken DNA ends [42]. An alternative NHEJ pathway that can repair DSBs with high fidelity has recently been proposed [43,44]. Because L1-EDSB hypermethylation is replication independent, these NHEJ pathways are candidates for methylated RIND-EDSB repair. While DNA-PKcs, a phosphatidylinositol-3-kinase, and Ku are required for the general NHEJ pathway, ataxia telangiectasia-mutated (ATM) acts jointly with checkpoint kinase 2 and BRCA1 to control the fidelity of DNA end-joining by precise NHEJ [44]. ATM and RAD51 are also important in homologous recombination repair of DNA damage [45].

The objective of this study was to evaluate whether EDSBs are processed differentially depending on the DNA methylation status of the surrounding genomic region. This information may explain why most DSBs are hazardous to cells, while significant numbers of methylated L1-EDSBs are universally present in all cell types including non-transformed/cancerous and do not lead to the same problems that other types of DSBs do. Moreover, if the degrees of repair precision for methylated and unmethylated L1-EDSBs are distinct, this mechanism may connect genomic hypomethylation and genomic instability.

## Results

### Detection of EDSBs in non-replicating cells

EDSB-PCR measures the number of unrepaired or modified EDSB ends at a specific time point. It does not chronologically visualize DNA breakage and repair processes. Therefore, each observation represents the outcome of EDSB production, retention, and repair combined. Since the sources of RIND-EDSBs are unknown, we assumed that, besides the independent variable of each experiment, other factors that may influence RIND-EDSBs in our experiments were the same between test and control cells grown under the same condition.

**Figure 2:**
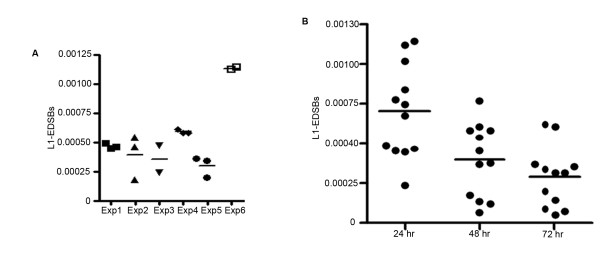
**Levels of L1-EDSBs**. The figures show the number of L1-EDSB genomes per genome digested with *Eco*RV and *Alu*I and ligated to the linkers or the number of L1-EDSB genomes per control genome. (A) Duplicates or triplicates of L1-EDSB quantification from different passages and incubation times in serum-free media. Each dot of the same experiment (exp) marks HeLa cells from the same passage but derived from different tissue culture flasks. Dots within the same drawing mark cells from different experiments but whose DNA and PCR experiments were prepared simultaneously. (B) L1-EDSBs incubated for different amounts of time, 24, 48 and 72 hrs, in serum-free media.

To analyze EDSBs present in non-replicating cells, we first evaluated the level of RIND-EDSBs by measuring the number of L1-EDSBs present under conditions of serum deprivation. The results show that L1-EDSBs were detectable in all samples (Fig. 2A). When cells from the same passage were separated and simultaneously cultured, we observed consistent levels of EDSBs in each experiment, suggesting that our measurements were precise and reproducible (Fig. 2A). There was no statistical difference in the number of EDSBs between samples incubated in serum-free media for 48 and 72 hrs (n = 12, two-tailed paired *t*-test, p = 0.0926) (Fig. 2B); however, levels of L1-EDSBs at 48 hrs were significantly lower than those at 24 hrs (n = 12, two-tailed paired *t*-test, p = 0.031) (Fig. 2B). There are 3388 LINE-1 primer homologs http://blast.ncbi.nlm.nih.gov. If the average EDSB PCR amplicon size is 300 bp, one L1-EDSB would represent approximately 2,200 EDSBs. By this estimation, cells under serum deprived condition possessed approximately 0.7 to 3.47 EDSBs per cell. This indicates that RIND-EDSBs were commonly produced in the absence of any agents known to cause DNA damage and that these RIND-EDSBs were being repaired during the course of our experiment.

### Replication-independent EDSB reduction by trichostatin A treatment

**Figure 3:**
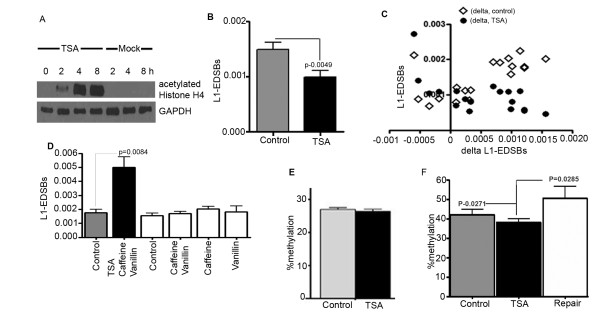
**L1-EDSBs and TSA**. (A) Immunoblot of acetylated histone H4 showing an increase in histone acetylation at 2 hrs after TSA treatment, saturation at 4 hrs and persistence up to 8 hrs. HeLa cells treated with TSA and vehicle control. (B) Comparison between L1-EDSBs of HeLa cells treated with TSA for 4 hrs and control cells. (C) Comparison of decreased L1-EDSBs on X axis and L1-EDSB levels of controls or tests on Y axis. Delta L1-EDSBs was decreased L1-EDSBs which was the levels of L1-EDSBs of control minus TSA. L1-EDSBs of control were ◇, and TSA were ● (D) Comparison between L1-EDSBs of HeLa cells treated with several combinations of TSA, caffeine and vanillin for 4 hrs and the control. (E) Comparison between COBRA-L1 analysis of control and TSA-treated cells. (F) Methylation levels of L1-EDSB of control, HeLa cells treated with TSA and repaired EDSBs, following the formula {(% methylation of L1-EDSB × L1-EDSBs) of control - (% methylation of L1-EDSB × L1-EDSBs) of test}/(L1-EDSB of control - L1-EDSB of test). Tests were HeLa cells treated with TSA. (B, C, E and F) Data represent means ± SEM.

We previously showed that EDSBs are hypermethylated [1]. Higher L1-EDSB methylation levels suggest that there are more unrepaired RIND-EDSBs near methylated genomic regions. Since DNA methylation is usually associated with histone deacetylation [46], we determined whether RIND-EDSBs would be repaired if the chromatin became hyperacetylated. We treated HeLa cells with a histone deacetylase inhibitor, Trichostatin (TSA), to hyperacetylate histones and consequently decondense the chromatin [47-49]. Histone acetylation was observed at 2 hrs, and the level peaked at 4 hrs (Fig. 3A). We compared the number of EDSBs in the control and in cells after 4 hrs of TSA treatment. TSA treatment of serum-deprived HeLa cells significantly reduced the number of L1-EDSBs (two-tailed paired *t*-test, n = 18, p = 0.0049) (Fig. 3B). Assuming that TSA did not prevent EDSB formation, this data suggests that RIND-EDSBs were retained prior to TSA treatment and that histone hyperacetylation facilitated RIND-EDSB repair.

Furthermore, we compared the numbers of L1-EDSBs of control and TSA-treated samples with the levels of L1-EDSB reduction (control - TSA treated) (Fig. 3C). We observed a strong direct correlation between the levels of L1-EDSB reduction and the number of L1-EDSBs of control cells (n = 14, Pearson r = 0.8471, p value (one-tailed) < 0.0001) (Fig. 3C). In contrast, there was no correlation between the levels of L1-EDSB reduction and the L1-EDSBs of TSA-treated samples (r = -0.2733, p = 0.1722) (Fig. 3C). This result indicates that control samples not only possess a larger number of RIND-EDSBs but also a wider range of EDSB levels. Moreover, each sample with hyperacetylated chromatin contained similar few numbers of RIND-EDSBs. This suggests that variable numbers of RIND-EDSBs maintained when chromatin is deacetylated. We concluded here that heterochromatin is a reservoir of RIND-EDSBs.

To examine the role of DSB repairs on RIND-EDSBs reduction by TSA treatment, we combined TSA treatment with inhibitors of critical NHEJ proteins; vanillin [50] and caffeine [51], inhibitors of DNA-PKcs and ATM, respectively. At 4 hrs, histones were hyperacetylated (data not shown). In contrast to TSA treatment alone, the number of L1-EDSBs was not reduced but increased (two = tailed paired t-test, n = 6, p = 0.0084) (Fig. 3D). In this combined treatment, even though TSA-induced histone hyperacetylation may expose retained RIND-EDSBs, NHEJ inhibitors may prevent the repair of these lesions. This suggests that the reduction of EDSBs in TSA-treated cells, as demonstrated in figure 3B, results from the function of NHEJ repair. Moreover, the difference in RIND-EDSB levels between TSA-treated and control cells (Fig. 3B) is not simply because the effect of TSA-induced hyperacetylation on chromatin structure could somehow affect breakage during DNA purification and lead to changes in the number of detected DSBs.

RIND-EDSBs increased when TSA treatment was combined with NHEJ inhibitors (Fig. 3D) suggesting that RIND-EDSBs can be produced. Similarly, sporadic increase in unmethylated EDSBs can be found when cells are cultured with vanillin for 24 hrs (Additional file 3). However, when treated with vanillin or caffeine or both without TSA for 4 hrs, although there were sporadic increments of RIND-EDSBs, these results were not statistically significant (Fig. 3D). Therefore, hyperacetylation-associated DNA may be prone to produce more RIND-EDSBs. This data may be similar to a number of reports that TSA increases low dose radiation sensitivity that TSA may increase DNA fragility [52-58].

We further analyzed the effect of TSA on the level of DNA methylation using COBRA-L1 assay [1]. TSA did not alter genomic LINE-1 methylation levels (Fig. 3E). However, we observed that the methylation level of L1-RIND-EDSBs of TSA-treated samples (Fig. 3B) was lower than that of controls (one-tailed paired *t*-test, n = 15, p = 0.0271). The percentage methylation levels of repaired EDSBs were calculated from the reduced EDSBs by TSA. The methylation level of L1-RIND-EDSBs of TSA-treated samples was also lower than repaired EDSBs (one-tailed paired *t*-test, n = 15, p = 0.0285) (Fig. 3F). This result suggests that retained RIND-EDSBs are more highly methylated. In addition, TSA treatment increases histone acetylation and consequently causes immediate repair (or end modification) of methylated L1-EDSBs.

### Replication-independent EDSBs and γ-H2AX

**Figure 4:**
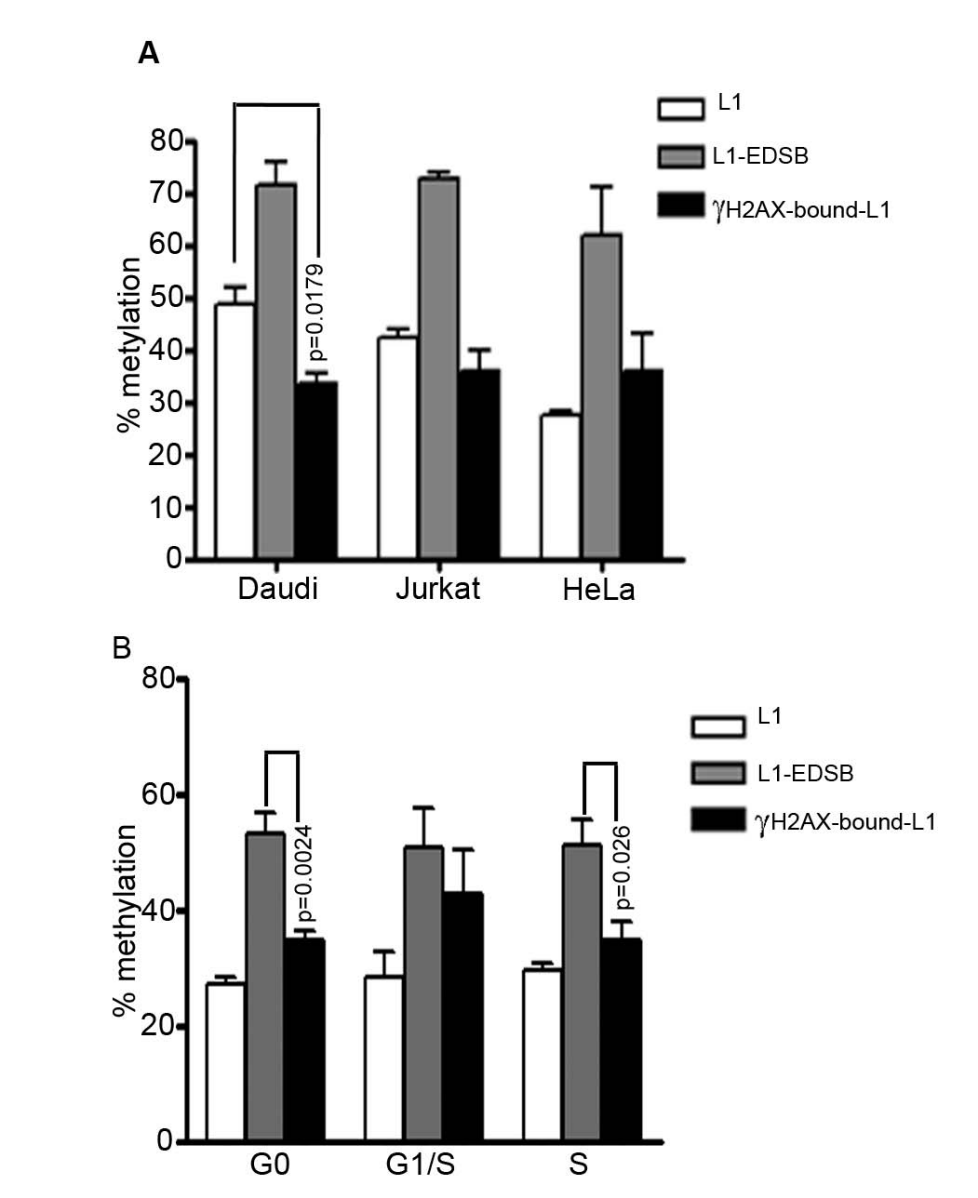
**Methylation statuses of γH2AX-bound LINE-1s**. (A, B) LINE-1 methylation levels of genomic LINE-1s, L1-EDSBs and γ-H2AX-bound LINE-1s in (A) Daudi, Jurkat and control HeLa cells and (B) in HeLa cells in the G0, G1/S and S phases. Data represent means ± SEM.

γ-H2AX foci are one of the earliest observable events in DSB repair responses [20]. While RIND-EDSBs are retained within heterochromatin, γ-H2AX foci form preferentially in euchromatin over heterochromatin after exposure to ionizing radiation [59]. Therefore, we investigated whether the relationship between RIND-EDSBs and γ-H2AX is reversed under our conditions. γ-H2AX-bound DNA was obtained by Chromatin Immunoprecipitation (ChIP) [60] using a γ-H2AX antibody, and bound LINE-1 sequences were quantified by real-time PCR using 5' LINE-1 primers [24] (Additional file 4 and 5). LINE-1s near RIND-EDSBs were consistently hypermethylated [1]. When we analyzed the methylation status of γ-H2AX-bound LINE-1s, we found that γ-H2AX-bound LINE-1s in all cells were significantly less methylated than L1-EDSBs (two-tailed paired *t*-test; n = 3; p = 0.008, 0.0193, 0.0243 for Daudi, Jurkat and HeLa cells, respectively) (Fig. 4A). The γ-H2AX-bound LINE-1s in Daudi cells were also significantly less methylated than genomic LINE-1s (p = 0.0179) (Fig. 4A). Moreover, in G0, there was a more prominent difference between the methylation levels of L1-EDSBs and γ-H2AX-bound LINE-1s (two-tailed paired *t*-test, n = 6, p = 0.0024) than in S phase (p = 0.026) (Fig. 4B). Therefore, a significant number of methylated LINE-1s near RIND-EDSBs may be free from γ-H2AX.

### γ-H2AX-bound DNA and histone acetylation

**Figure 5:**
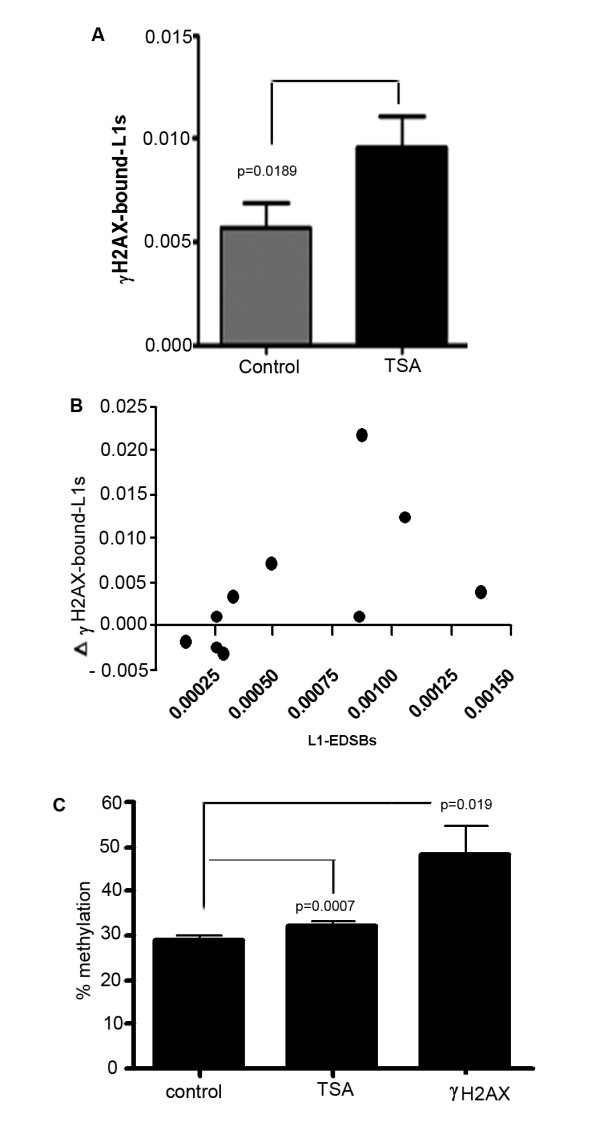
**γH2AX-bound LINE-1s in cells treated with TSA or left untreated**. (A) γ-H2AX-bound LINE-1 genomes per cell treated with TSA and per control cell. (B) Correlation between the increased levels of γ-H2AX-bound L1s and L1-EDSB of control. ΔγH2AX-bound-L1s were increased γ-H2AX-bound L1s levels, calculated by the levels of TSA minus control, and L1-EDSB of controls were L1-EDSB genomes per control genome of HeLa prior to TSA treatment. (C) Methylation levels of γH2AX-bound LINE-1s of control, TSA-treated HeLa and increased γH2AX-bound DNA after TSA treatment (ΔγH2AX). Percent methylation of ΔγH2AX was calculated using the following formula: ((%methylation X γH2AX-bound LINE-1s of TSA) - (%methylation X γH2AX-bound LINE-1s of control))/((γH2AX-bound LINE-1s of TSA) - (γH2AX-bound LINE-1s of control)). The control group was comprised of cells treated with solvent lacking TSA. Data represent means ± SEM.

We further explored if there is a reduction in H2AX phosphorylation around heterochromatin related RIND-EDSBs. In contrast to its effect on the number of RIND-EDSBs, TSA increased the number of γ-H2AX-bound LINE-1s (two-tailed paired *t*-test, n = 16, p = 0.0189) (Fig. 5A). These data indicate that RIND-EDSBs are retained in heterochromatin and remain unbound by γ-H2AX. When histones become hyperacetylated, retained RIND-EDSBs may be exposed and consequently undergo H2AX phosphorylation. The increase in γ-H2AX-bound LINE-1s was directly correlated with the number of L1-EDSBs that existed prior to the beginning of the experiment (n = 10, Spearman r = 0.7576, p value (two-tailed) = 0.0149) (Fig. 5B). Therefore, the level of TSA-induced increase in γ-H2AX depends on the number of retained RIND-EDSBs. This finding that TSA treated cells had more γ-H2AX bound DNA is similar to a report that γ-H2AX foci form preferentially in euchromatin but not in heterochromatin after exposure to ionizing radiation [59].

We further examined the methylation levels of γ-H2AX-bound LINE-1s after TSA treatment. TSA-treated samples with increased numbers of γ-H2AX-bound LINE-1s possessed higher levels of γ-H2AX-bound LINE-1 methylation than controls (two-tailed paired *t*-test, n = 8, p = 0.0007) (Fig. 5C). This higher methylation level was due to the process by which histone hyperacetylation allowed new γ-H2AXs to be produced on methylated genomes. The methylation levels of increased γ-H2AX-bound LINE-1s (Δγ-H2AX) were also higher than in the control (p = 0.019) and in TSA-treated samples (p = 0.0447) (Fig. 5C). These changes in γ-H2AX-bound LINE-1 methylation levels by TSA supported the hypothesis that retained methylated RIND-EDSBs are devoid of γ-H2AX.

### Methylation-dependent differential repair pathways of replication-independent EDSBs

**Figure 6:**
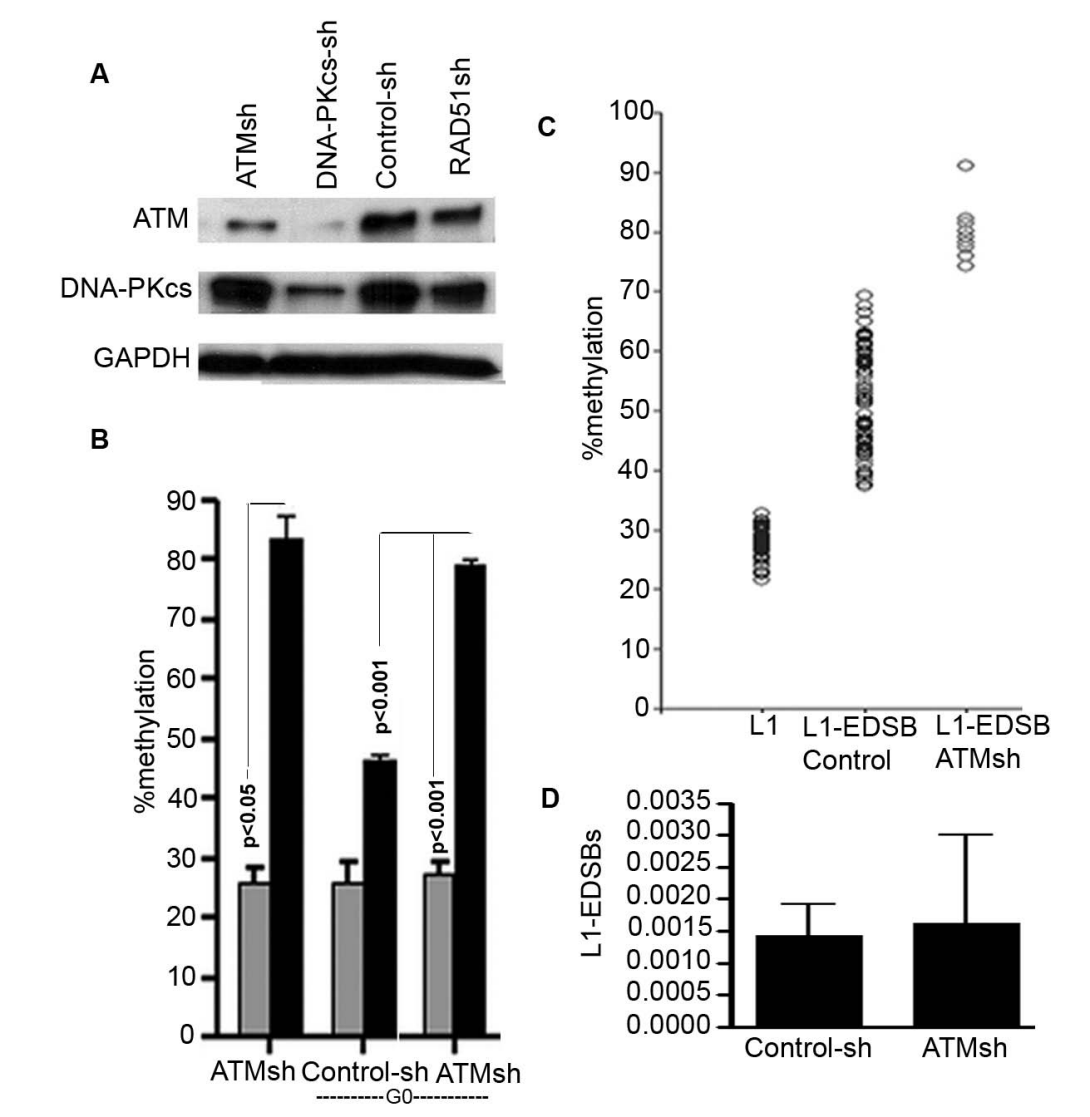
**Methylated EDSBs may be repaired by an ATM-dependent pathway**. (A) Immunoblots of ATM and DNA-PKcs in ATM shRNA-transfected HeLa cells. GAPDH is included as a loading control. (B) methylation of L1 and methylation of L1-EDSB analysis of ATM shRNA-transfected HeLa cells. (C) methylation of L1 and methylation of L1-EDSB analysis of ATM shRNA- and control shRNA-transfected HeLa cells. The level of EDSB methylation of ATM shRNA-transfected cells was higher than EDSBs of all tests in this and a previous study (1). Each circle represents an individual methylation of L1 or L1-EDSB result. (D) Levels of L1-EDSBs. The data represent the number of L1-EDSB genomes per genome digested with *Eco*RV and *Alu*I and ligated to the linkers or the number of L1-EDSB genomes per control genome. (B) and (D) Data represent means ± SEM, with statistical significance determined by two-tailed paired *t*-test.

Since methylated L1-EDSBs are retained under normal physiological conditions, methylated L1-EDSBs may be repaired via a biological pathway that is different from that used for the repair of unmethylated L1-EDSBs [1]. We therefore analyzed L1-EDSB methylation levels in cells expressing short hairpin RNA targeting ATM, DNA-PKcs, Ku86 and RAD51, which are required for NHEJ or homologous recombination repair (Fig. 6 and Additional file 6). We chose to use specific shRNAs to perturb the respective repair pathways because genomic LINE-1 methylation levels vary widely in different cell types [4,61]. In this way, we were able to examine the effects of each repair pathway in the same epigenetic background with the fewest possible confounding factors.

DSBs can be repaired by several pathways [33]. Inhibition of a particular pathway will increase L1-EDSB methylation levels if that pathway is responsible for the repair of methylated L1-EDSBs and if other pathways cannot compensate. Cells with ATM knocked down (Fig. 6A) cultured in serum-free media had markedly increased L1-EDSB methylation levels (Fig. 6B). There are several DSB repair pathways [41-45,62-65], and they can be employed interchangeably for radiation-induced DSBs [64,66,67]. In contrast, our results demonstrate that methylated L1-EDSB repair is ATM-dependent and there is no compensatory pathway. Stable transfection of HeLa cells with DNA-PKcs shRNA caused down-regulation of not only DNA-PKcs, but also ATM (Fig. 6A), as has previously been observed [68]. Therefore, the effects of DNA-PKcs knockdown were not evaluated. L1-EDSB methylation levels in cells treated with shRNA for Ku86, a DNA-PKcs-dependent NHEJ pathway protein, and RAD51, a homologous recombination repair dependent protein, were similar to the methylation levels in the control (Additional file 6). Therefore, in contrast to the loss of ATM, the inhibition of the DNA-PK-dependent NHEJ pathway and inhibition of homologous recombination repair did not result in an increase in L1-EDSB methylation, illustrating that these pathways play a lesser role in the repair of methylated L1-EDSBs. The lack of accumulation of unmethylated L1-EDSBs may be the result of several mechanisms that are involved in the repair of radiation induced DSBs and may repair unmethylated L1-EDSBs. The specificity of ATM-dependent methylated EDSB repair was confirmed when HeLa L1-EDSB methylation levels from up to 100 tests were lower than those for cells treated with shRNA targeting ATM (Fig. 6C). In conclusion, these results suggest that methylated and unmethylated L1-EDSBs are repaired preferentially by different pathways. Under non-replicating conditions, methylated L1-EDSBs are selectively repaired by the ATM-dependent end-joining pathway. However, the number of L1-EDSBs between ATM knockdown cells and controls were not different (Fig. 6D). This may imply that the production or repair of unmethylated L1-EDSBs may be related to the number of retained methylated L1-EDSBs.

## Discussion

### Replication-independent EDSB production, retention and repair rates

**Figure 7:**
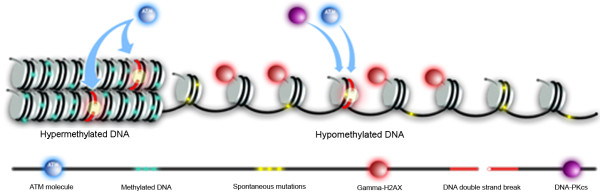
**Sequences nearby RIND-EDSBs are hypermethylated, and RIND-EDSBs are retained in heterochromatin and preferentially repaired by different pathways**. A diagrammatic representation of RIND-EDSBs under normal physiological conditions showing the differences between hyper- and hypomethylated DNA, which associate with hetero- and euchromatin [48], respectively. RIND-EDSBs are frequently present near methylated DNA [1]. While methylated L1-RIND-EDSBs are concealed in heterochromatin, the earliest DSB repair response, γ-H2AX deposition on chromatin, is more prevalent in hypomethylated DNA. The differential NHEJ repair pathways in non-replicating cells between hyper- and hypomethylated DNA are shown. ATM-mediated end-joining repair prefers methylated EDSBs and more precisely repairs breaks than other mechanisms [42]. NHEJ pathways at hypomethylated genomes may be similar to the processes that repair radiation-induced DSBs in that the repair processes are interchangeable [64,66,67]. Other error-prone, less known and redundant pathways are not included in the diagram. However, these pathways may be prevented from repairing methylated RIND-EDSBs. Spontaneous mutations accumulate more quickly in hypomethylated regions of the genome [9,10,15].

In this study, we report evidence for the existence of replication-independent EDSBs that are hypermethylated and likely retained preferentially in heterochromatin. We hypothesize that RIND-EDSBs are hypermethylated because there is a time lag between the production and the repair of methylated L1-EDSBs and thus unrepaired, un-modified EDSB ends can be detected as RIND-EDSB retention (Fig. 7). We showed that when chromatins become hyperacetylated the numbers of RIND-EDSBs were reduced. This not only suggests that compact chromatin is associated with EDSB retention, but also that euchromatin may associate with faster EDSB repair processes. Moreover, when methylated RIND-EDSB repair was inhibited by ATM shRNA or caffeine alone, the levels of RIND-EDSB were not increased. Therefore, the activity of heterochromatin or methylated chromatin-associated RIND-EDSB production should be low.

An alternative, but less preferable, hypothesis for our observations would be that there is no EDSB retention. In this scenario, unmethylated or euchromatin-associated DNA is stable, while methylated DNA is more fragile, so TSA may limit the production of methylated EDSB. When Ku dependent NHEJ was inhibited, sporadic increase in unmethylated EDSBs can be observed (Additional file 3). Moreover, EDSBs immediately increased when DSB repair was inhibited in cells treated with TSA. Therefore, unmethylated EDSBs can be produced, particularly more efficiently at hyperacetylated chromatin. It is also unlikely that methylated DNA is broken faster than repaired or there is no methylated EDSB repair. If this hypothesis was true, we would have observed a continuous increase in EDSB methylation levels, no higher EDSB methylation level in ATMsh cells and instability of DNA methylation.

### Replication-independent EDSB retention and γ-H2AX

Histone hyperacetylation reduced the number of RIND-EDSBs and increased the amount of methylated γ-H2AX-bound DNA. Moreover, whereas L1-EDSBs were hypermethylated, methylation levels of γ-H2AX-bound LINE-1s were lower, and in some cases lower than the genomic level. Therefore, in contrast to euchromatin-related RIND-EDSBs, methylated RIND-EDSBs are likely retained in heterochromatin where the compacted structure prevents conventional cellular DSB responses, such as H2AX phosphorylation (Fig. 7).

RIND-EDSB retention may be the opposite of what is generally believed for DSBs. DSBs are known to be hazardous events. Even a single DSB, if unrepaired, will induce lethality [69]. However, it is reasonable to find that EDSBs are retained when the DSB ends are shielded from general cellular responses to DSBs. For example, signal EDSB ends can persist within V(D)J recombination complexes and do not normally activate the DNA damage-dependent cell cycle checkpoint [70]. We speculate that cellular responses to retained RIND-EDSBs may be delayed by the chromatin conformation. DNA methylation is usually associated with heterochromatin [71], whose tightly packed structure may brace the broken chromosome. Recently, Cowell *et al*. found that γ-H2AX foci form preferentially in euchromatin but not in heterochromatin after exposure to ionizing radiation [59]. In S phase, EDSBs are still hypermethylated, albeit less significantly than in the G0 phase [1]. Because DNA replication does not occur simultaneously throughout the genome, heterochromatin may capture the RIND-EDSBs located far from replication forks.

There are several scientific findings surrounding DNA breakage and repair that have not yet been explained, and RIND-EDSB retention may help provide further insight into these unexplained phenomena. A few examples are discussed here. First, single-cell PCR is an important method for preimplantation diagnosis [72], but allele drop out is a major drawback of this technique [73]. This could be explained if RIND-EDSBs are present in the PCR template of one allele and so could lead to a drop out of that allele. Second, histone deacetylase inhibitors have been found to induce γ-H2AX deposition in several cancer types, especially leukemia [74]. This is similar to our observations and it would be interesting to evaluate RIND-EDSB retention in leukemic cells. Interestingly, in 2005, Yaneva *et al*. reported high cellular toxicity when NHEJ inhibitors and TSA were combined [75]. It would be important to further determine if this toxicity was facilitated by the increase in euchromatin-associated RIND-EDSB, and consequently are more sensitive to NHEJ inhibitors. Thirdly, several environmental and genetic conditions can result in γ-H2AX deposition on chromatin, however, it is unclear if these conditions induce DNA breaks [19,22-30]. Finally, global hypomethylation was not only found in cancer but also during aging [76]. However, P53 mutation not only prevents cell death from DNA breaks but also contributes to immortalization, an opposite phenotype from aging [77]. It would be interesting to further explore the role of EDSBs under these conditions.

### Replication-independent EDSB production

Radiation-induced DSBs are hazardous to cells and can lead to faulty DNA recombination. Therefore, production of RIND-EDSBs in all cells in the absence of strong environmental insults and apoptotic induction warrants investigation. Even though L1-EDSBs were significantly reduced during prolonged cell culture in G0, increased levels of L1-EDSBs from matched samples were sometimes observed. L1-EDSBs were found more frequently when cells were cultured with a DNA-PKcs inhibitor (Additional file 3) or a combination of TSA and NHEJ inhibitors (Fig. 3D). This suggests that RIND-EDSBs can be produced without chemical- or radiation-induced DNA breakage. The precise mechanisms that produce RIND-EDSBs are unknown. Several types of cellular stress, including temperature, osmolarity, oxidative DNA damage and endonucleases [22-29], result in γ-H2AX foci. However, DSBs do not play a role in heat- or hypertonicity-induced cell death [26,34]. Interestingly, down-regulation of several genes that do not directly control DNA replication or the cell cycle but are involved in DNA binding, ion flux, gene regulation and RNA processing also increases γ-H2AX foci [30]. Therefore, it is possible that many cellular phenomena besides DNA replication produce EDSBs.

### Connection between global hypomethylation and genomic instability

Genomic instability is a cardinal feature of cancer [78]. Understanding the molecular mechanisms involved in this instability is essential for the development of effective approaches in cancer prevention [79] and treatment to prevent cancer progression [78]. RIND-EDSBs may mediate mutations that are produced by genomic hypomethylation. Hypomethylation-induced mutations are the result of recombination between different loci. Under normal condition, RIND-EDSBs are hypermethylated; therefore, the mechanical DNA repair processes for methylated and unmethylated L1-EDSBs should be different. We found a remarkable increase in RIND-EDSBs when chromatin became hyperacetylated and NHEJ repair was inhibited at the same time. Therefore, euchromatin-associated DNA may be prone to be broken, but unmethylated L1-EDSBs may be immediately repaired. In general, DSB repair pathways are redundant and interchangeable [66], but reduced ATM expression leads to increased methylation of L1-EDSBs (Fig. 7). Retained RIND-EDSBs may be similar to radiation-induced DSBs in heterochromatin that are slowly repaired by ATM [80]. In contrast to other NHEJ pathways, the ATM-dependent repair pathway has been proposed to be more precise [43]. Therefore, methylated L1-EDSBs, but not unmethylated forms, may be able to escape error-prone NHEJ repair. Consequently, the rate of spontaneous mutations in methylated DNA may be less than in hypomethylated genomic regions (Fig. 7). In cancer, DNA is globally hypomethylated, consequently, more EDSBs may be repaired by the more error-prone pathways which could lead to genetic instability, higher mutation rate.

## Conclusion

Our results show that L1-EDSBs are detectable and hypermethylated in non-replicating cells, and that RIND-EDSBs in methylated genomic regions are likely retained in heterochromatin. Unlike radiation-induced DSBs and euchromatin-associated RIND-EDSBs, retained methylated RIND-EDSBs do not initiate an immediate cellular DNA damage response, which can lead to fast but more error-prone repair or to cell death. Moreover, our data suggest that retained RIND-EDSBs are slowly repaired by the more precise ATM-dependent DSB repair pathways. This process may help prevent spontaneous mutations within methylated genomic regions and consequently, hypomethylated genome in cancer is mutated faster than methylated DNA (Fig. 7).

## Methods

### Cell culture

The cell lines used were HeLa (cervical cancer), Daudi (B lymphoblast) and Jurkat (T cell leukemia). To inhibit DNA replication, the cells were cultured in serum deprivation medium for 48 hr. HeLa cells in G1/S and S phase were synchronized by the thymidine block method and were cultured with 2 mM thymidine (Sigma-Aldrich, St. Louis, MO, USA) to obtain cells in the G1/S phase [81]. Flow cytometry was used to determine the stages of the cell cycle, as well as to identify fragmented and apoptotic cells. To evaluate the consequences of histone hyperacetylation, a single dose of 100 ng/ml TSA (Sigma-Aldrich), an inhibitor of histone deacetylase, was added to synchronized HeLa cells that had been deprived of serum for 48 hours. TSA was added for 2, 4 and 8 hours as indicated with or without 2.5 mM vanillin (Sigma-Aldrich) and 5 mM caffeine (Sigma-Aldrich). HeLa cells were treated for 24 hours with 2.5 mM vanillin. For radiation treatment, the medium of the HeLa cells was replaced with ice-cold medium, and the cells were exposed to 0.01, 0.1, 1.0, 2.0, 10, 20, 40, and 60 Gy γ-rays at a rate of 6.22 cGy/min with a ^60^Co source (Eldorado78).

### High molecular weight DNA preparation

High molecular weight (HMW) DNA was prepared as described previously [1]. To prepare HMW DNA, 1× 10^6 ^cells were embedded in 1% low-melting-point agarose, lysed, and digested in 400 μl of 1 mg/ml proteinase K, 50 mM Tris, pH 8.0, 20 mM EDTA, 1% sodium lauryl sarcosine. The plugs were rinsed four times in TE buffer for 20 min. To polish cohesive-end EDSBs, T4 DNA polymerase (New England Biolabs, Beverly, MA, USA) was added, followed by four rinses in TE buffer for 20 min. The modified ligation mediated PCR (LMPCR) linkers were prepared from the oligonucleotides 5'-AGGTAACGAGTCAGACCACCGATCGCTCGGAAGCTTACCTCGTGGACGT-3' and 5'-ACGTCCACGAG-3'. The linkers (50 pmol) were ligated to HMW DNA using T4 DNA ligase (New England Biolabs) at 25°C overnight (fig. 1). DNA was extracted from the agarose plugs using a QIAquick gel extraction kit (QIAGEN, Basel, Switzerland).

### Detection and quantification of L1-EDSBs

A schematic representation of EDSB PCR is provided in figure 1b. After the LMPCR linkers were ligated to HMW DNA, the number of L1-EDSBs was measured as previously described for EDSB PCR with modifications as follows [1]. Duplicate or triplicate numbers of L1-EDSBs were measured by real-time PCR using an ABI PRISM^® ^7500 instrument (Applied Biosystems, Carlsbad, CA, USA) with LINE-1 primers 5'-CTCCCAGCGTGAGCGAC-3' (outward), the linker primer 5'-AGGTAACGAGTCA GACCACCGA-3' and the Taqman probe homologous to the 3' linker sequence (6-fam) ACGTCCACGAGGTAAGCTTCCGAGCGA (tamra) (phosphate). Amplification was performed with 0.5 μM of each primer, 0.3 μM Taqman probe, 0.025 U of HotStarTaq (QIAGEN, Valencia, CA, USA), 1× TaqMan^® ^Universal PCR Master Mix (Applied Biosystem) and 30 ng of ligated DNA for up to 60 cycles, with quantification after the extension step. Control HeLa DNA was digested with *Eco*RV and *Alu*I and ligated to the LMPCR linkers. The numbers of EDSBs were compared with the ligated control digested DNA and reported as LINE-1 ligated *Eco*RV and *Alu*I digested genome (L1-EDSBs) per cell. L1-EDSBs do not report exact number of EDSBs. EDSB PCR detects EDSBs within PCR efficiency from interspersed repetitive sequences to EDSB sequences. The number of L1-EDSBs depends on the number of LINE-1 sequences that can be hybridized by the LINE-1 primer under the PCR condition and the size of the PCR amplicons.

### Study of genomic LINE-1 and L1-EDSB methylation

We used combined bisulfate restriction analysis of LINE-1 (COBRA-L1) [4] to measure the methylation levels of genomic LINE-1s, and we used COBRA-L1 analysis of the LMPCR linker to measure LINE-1 methylation located near EDSBs (this method is referred to as COBRA-L1-EDSB) [1]. A schematic comparison of the COBRA-L1-EDSB and COBRA-L1 templates is provided in figure 1b. Ligated HMW DNA was modified with bisulfite. Bisulfite-modified DNA was recovered using a Wizard DNA clean-up kit (Promega, Madison, WI, USA) and desulfonated before PCR amplification. For COBRA-L1, bisulfate-treated DNA was subjected to 35 PCR cycles with two primers, B-L1-inward 5'-CGTAAGGGGTTAGGGAGTTTTT-3' and B-L1-outward 5'-RTAAAACCCTCCRAACCAAA TATAAA-3'. A hot-stop technique was used to prevent heteroduplex amplicons. The α^32^P-labeled-bisulfite-L1-outward oligo was added in the last PCR cycle. The amplicons were doubly digested in a 10 μl reaction volume with 2 U of *Taq*I and 8 U of *Tas*I in 1× *Taq*I buffer (MBI Fermentas, Vilnius, Lithuania) at 65°C for 4 hr. This method was designed to detect unmethylated and methylated sequences of 98 and 80 bp, respectively. The intensity of DNA fragments was measured with a PhosphorImager using Image Quant software (Molecular Dynamics, GE Healthcare, Slough, UK). The LINE-1 methylation level was calculated as the percentage of *Taq*I intensity divided by the sum of *Taq*I- and *Tas*I-positive amplicons. For COBRA-L1-EDSB, the B-L1-inward oligo was replaced with the B-LMPCR oligo, 5'-GTTTGGAAGTTTATTTTGTGGAT-3', and 40 PCR cycles were carried out according to the same protocol. Bisulfite-treated Daudi, Jurkat, and HeLa DNA digested with *Eco*RV and *Alu*I and ligated LMPCR linker were used as positive controls to normalize the inter-assay variation of all COBRA experiments. HeLa DNA without ligation was used as a negative control.

### shRNA

The oligonucleotide sequences of the shRNA targeting ATM and Rad51 have been previously described by Zhang, et al [82], DNA-PKcs by An, et al [83] and Ku86 by Wanninger et al [84]. Controls were and nonsilencing siRNA control oligoes with no homology to any known mammalian genes (Ambion, Austin, Texas, USA). These oligonucleotides were inserted into the PsilencerTM 3.1 vector (Ambion, Austin, Texas, USA) and transfection was mediated by siPORTTM *XP-1 *(Ambion, Austin, Texas, USA).

### Western blot analysis

Antibodies used for Western blots included an anti-GAPDH antibody (Trevigen, Gaithersburg, MD, USA) as a control; an antibody against acetylated-histone H4 that recognizes histone H4 acetylated at lysines 5, 8, 12 or 16 (Upstate, Charlottesville, VA, USA) for the analysis histone acetylation in TSA-treated cells; DNA-PKcs (G-4) (Santa Cruz Biotechnology, Santa Cruz, CA, USA), ATM (2C1) (GeneTex, San Antonia, Tx, USA), Ku86 (M20) (Santa Cruz Biotechnology) and Rad51 (H-92) (Santa Cruz Biotechnology) for the analyses of DNA-PKcs, ATM and Ku86 levels. In shRNA experiments the following antibodies were used: horseradish peroxidase (HRP)-goat anti-rabbit IgG (H+L) conjugate (Zymed^® ^Laboratories, San Francisco, CA, USA) for GAPDH and acetylated-histone H4 and goat anti-mouse IgG-HRP sc-2005 HRP conjugated (Santa Cruz Biotechnology) for ATM, DNA-PKcs and Ku86. Signals were developed with the Supersignal west chemiluminescent substrate optimization kit (Pierce, Rockford, IL, USA).

### ChIP

The ChIP assay was performed essentially as previously described with some modifications [24,60]. The chromatin fragments were immunoprecipitated with anti-phospho-Histone H2AX monoclonal antibody (Upstate, Charlottesville, VA, USA) or normal mouse IgG antibody as a negative control (Santa Cruz Biotechnology). Quantification of the amount of immunoprecipitated DNA was carried out by real-time 5'L1PCR using a QuantiTect SYBR Green PCR Kit (Qiagen, Basel, Switzerland) between the forward primer (L1.2HpaIIRFLPF: 5'-CTCCCAGCGTGAGCG AC-3') and reverse primer (5'LIDSIP1st: 5'-ACTCCCTAGTGAGATGAACCCG-3') located at the 5' end of LINE-1. The amount of γ-H2AX-bound LINE-1 sequences was used to calculate the quantity of precipitated genomic DNA by relating the LINE-1 quantity to the LINE-1s quantity of HeLa genomic DNA. The relative quantity unit was γ-H2AX-bound genome per cell. The precipitated DNA was then subjected to COBRA-L1.

### Statistical analyses

Statistical significance was determined according to a paired sample *t*-test or Pearson rank correlation statistics, when appropriate.

## List of abbreviations used

EDSBs: endogenous DNA double-strand breaks; LINE-1 or L1: long interspersed element-1; RIND-EDSBs: replication independent EDSBs; ATM: ataxia telangiectasia mutated; NHEJ: non-homologous end joining repair; DSBs: DNA double strand breaks; LMPCR: ligation-mediated polymerase chain reaction; TSA: Trichostatin; HMW: High molecular weight; COBRA: combined bisulfite restriction analysis; ChIP: Chromatin immunoprecipitation.

## Competing interests

The authors declare that they have no competing interests.

## Authors' contributions

NK carried out baselines and TSA treatment for PCR and ChIP experiments and performed the statistical analysis. CP, AT and PR participated in the baselines and TSA treatment for PCR and ChIP experiments, WaP participated in the shRNA for PCR experiments, WiP carried out the shRNA and vanillin treatment for PCR experiments, and AM conceived of and designed the study, interpreted data and wrote the manuscript. All authors read and approved the final manuscript.

## Supplementary Material

Additional file 1 Table summary 15 experiments from Pornthanakasem et al *Nucleic Acids Res *2008, **36**(11):3667-3675. Click here for file

Additional file 2 EDSB hypermethylation is DNA replication independent. Click here for file

Additional file 3 Changes in the quantity and methylation level of EDSBs after incubation with vanillin. Click here for file

Additional file 4 γ-H2AX-bound LINE-1s and radiation. Click here for file

Additional file 5 γ-H2AX-bound LINE-1s in several cells and cell cycles. Click here for file

Additional file 6 L1-RIND-EDSB methylation statuses of Ku86si and Rad51si cells. Click here for file
