# Supplementary material for: Replication independent DNA double-strand break retention may prevent genomic instability
Source: Mol Cancer. 2010 Mar 31;9:70. doi: 10.1186/1476-4598-9-70 (PMC2867818; doi:10.1186/1476-4598-9-70)
Supplement: Additional file 1 — Table summary 15 experiments from Pornthanakasem et al Nucleic Acids Res 2008, 36(11):3667-3675. [file 1476-4598-9-70-S1.PDF]

Additional file 1

Table summary 15 experiments from Pornthanakasem et al *Nucleic Acids Res* 2008, 36(11):3667-3675

	Experiment	Result	Conclusion
1.	EDSB PCR of several cancer cell lines, peripheral white blood cells and sperm.	Significant amounts of EDSBs, approximately 0.5 to 10 EDSBs per cell, were detected in all samples, including cancer cell lines, sperm cells and WBCs from several individuals.	Small and variable amounts of EDSBs are always present.
2.	EDSB PCR analysis of DNA from HeLa cells using different DNA-extraction methods: DNA prepared in gel, liquid DNA, liquid DNA from gel-embedded high molecular weight (HMW) DNA, and liquid DNA embedded in gel following the HMW DNA preparation protocol.	HMW DNA yielded the lowest amount of L1-DSBs.<br>Combined DNA preparations yielded an amount of L1-DSBs close to that of liquid DNA.	HMW DNA preparation does not induced DSBs.
3.	EDSB PCR on several cancer cell lines using L1-inward and L1-outward primers.	Similar L1-EDSB levels were observed regardless of the nature of the linked EDSB sequences, L1 sequences or unique sequences.	EDSBs are present both outside and inside LINE-1 sequences.
4.	EDSB PCR analysis of several cancer cell lines using L1, Alu and Tigger1 primers.	Numbers of EDSBs were in direct proportion to interspersed repetitive sequence type copy numbers in the human genomes.	EDSBs are randomly and widely distributed.
5.	EDSB PCR of HMW DNA from HeLa cells with and without T4 polymerase treatment.	Blunt and polished-end EDSBs from HeLa cells were identified.	EDSB ends are heterogeneous and the majority is blunt.
6.	EDSB PCR and the proportion of fragmented cells of HMW DNA from HeLa cells, Daudi cells and Daudi cells treated with DNase were measured.	The number of L1-EDSBs was independent of the proportion of fragmented cells.	The number of EDSBs is not related to the proportion of fragmented cells.
7.	EDSB PCR of HMW DNA from irradiated HeLa cells.	The number of L1-EDSBs was immediately increased by irradiation.	EDSB PCR can quantify DSBs.
8.	EDSB PCR of HMW DNA	L1-EDSBs were	EDSBs are produced in all

	from several cell cycles.	identified in all cell phases, with G0 having the lowest number.	cell phases including non-replicating phases.
9.	Combine bisulfate restriction analysis of LINE-1 (COBRA-L1) and COBRA-L1-EDSB of the mixture of HeLa and Daudi DNA digested with *Alu*I and *Eco*RV and ligated to an LMPCR linker.	Similar methylation levels were observed.	COBRA-L1-EDSB precisely measures LINE-1 methylation levels, and the levels can be directly compared with COBRA-L1 results.
10.	COBRA-L1 and COBRA-L1-EDSB of DNA from HeLa cells using different DNA-extraction methods: DNA prepared in gel, liquid DNA, liquid DNA from gel-embedded HMW DNA and liquid DNA into gel following by HMW DNA preparation protocol.	HMW DNA yielded the highest L1-DSB methylation levels.<br>Combined DNA preparations yielded methylation levels of L1-DSBs close to those for liquid DNA.	LINE-1 sequences near DNA extraction procedure-induced DSBs are less methylated than L1-EDSBs.<br>HMW DNA may not produce, or may produce significantly less, DNA extraction procedure induced-DSBs.
11.	COBRA-L1 and COBRA-L1-EDSB of HMW DNA from several cancer cell lines, peripheral white blood cells and sperm.	COBRA-L1-EDSB levels were always higher than COBRA-L1 levels.	L1-EDSBs possess higher methylation levels than the rest of the genomic LINE-1s.
12.	Southern blot hybridization of *Hpa*II- and MspI-digested DNA templates with an L1-EDSB-LMPCR probe or with an experimentally induced methylation-independent L1-DSB control probe.	The L1-EDSB-LMPCR probe hybridized to larger fragments of HpaII-digested DNA.	1. EDSBs are located within heavily methylated genomic regions.<br>2. Methylation near EDSB sequences preexisted in genome before DNA breakage process occurs.<br>3. This experiment validated the results for COBRA-L1 and COBRA-L1-EDSB analysis from experiment 11.
13.	COBRA-L1 and COBRA-L1-EDSB of HMW DNA from several cell cycles.	G0 cells contained the most significant hypermethylation level of L1-EDSBs.	The L1-EDSB hypermethylation mechanism is DNA replication independent.
14.	COBRA-L1 and COBRA-L1-EDSB of apoptotic DNA	Apoptotic DNA possessed genomic methylation level. Apoptotic DNA methylation was not measurable by COBRA-L1-EDSB.	Hypermethylation of L1-EDSB is not a result of apoptotic DNA fragmentation. Majority of L1-EDSB-LMPCR is not derived from apoptotic DNA.
15.	Detect apoptotic DNA by realtime LMPCR	No positive result	EDSB-LMPCR positive result was not from apoptotic DNA.

Note: We demonstrated that, first, high molecular weight (HMW) DNA preparations produce minimal or insignificant DNA breakage as detected by EDSB PCR. Second, the L1-EDSB methylation levels are higher than genomic L1s as detected by COBRA-L1-EDSB in comparison with COBRA-L1. Third, the hypermethylation of L1-EDSB was validated by Southern blot hybridization to an *Hpa*II-digested genome template using a labeled L1-EDSB-LMPCR probe. Finally, we also found that higher levels of EDSB methylation were not a characteristic of apoptotic DNA and DNA preparation-induced DSBs (1).

1. Pornthanakasem, W., Kongruttanachok, N., Phuangphairoj, C., Suyarnsestakorn, C., Sanghangthum, T., Oonsiri, S., Ponyeam, W., Thanasupawat, T., Matangkasombut, O., and Mutirangura, A. (2008) *Nucleic Acids Res* **36**, 3667-3675
