# Supplementary material for: Replication independent DNA double-strand break retention may prevent genomic instability
Source: Mol Cancer. 2010 Mar 31;9:70. doi: 10.1186/1476-4598-9-70 (PMC2867818; doi:10.1186/1476-4598-9-70)
Supplement: Additional file 2 — EDSB hypermethylation is DNA replication independent. [file 1476-4598-9-70-S2.PDF]

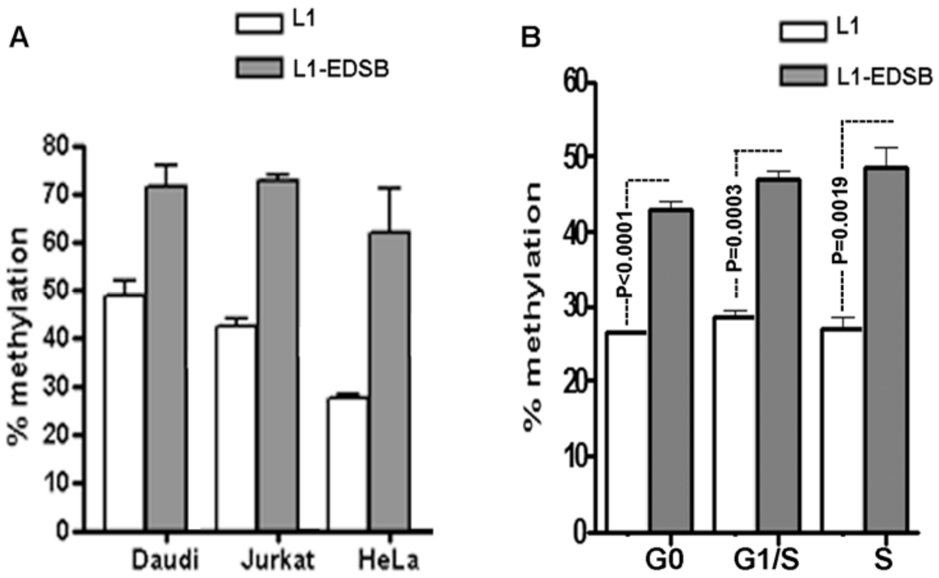

Additional file 2

EDSB hypermethylation is DNA replication independent.

(A) Methylation levels of L1 and L1-EDSB in Daudi, Jurkat and HeLa cells. (B) HeLa cells were synchronized at G0 by culture in serum-free medium for 48 hrs. HeLa cells in G1/S and S phases were synchronized by the thymidine block method with 2 mM thymidine to obtain cells at G1/S phase. COBRA-L1 and COBRA-L1-EDSB of HeLa cells at G0, G1/S and 3 hrs after the release into S phase from thymidine block. G0 had the most significant hypermethylation level of EDSBs. Data represent means ±SEM, with statistical significance determined by a two-tailed paired *t*-test.
