# Supplementary material for: Replication independent DNA double-strand break retention may prevent genomic instability
Source: Mol Cancer. 2010 Mar 31;9:70. doi: 10.1186/1476-4598-9-70 (PMC2867818; doi:10.1186/1476-4598-9-70)
Supplement: Additional file 3 — Changes in the quantity and methylation level of EDSBs after incubation with vanillin. [file 1476-4598-9-70-S3.PDF]

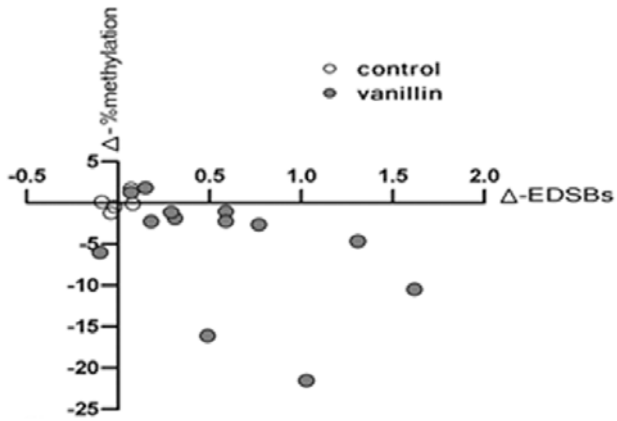

Additional file 3

Changes in the quantity and methylation level of EDSBs after incubation with vanillin

The Δ-EDSB axis x 10^{-3} and the Δ-%methylation axis are EDSB quantities and methylation levels of vanillin-treated cells corrected for background, respectively. This experiment indicated that RIND-EDSB production is not methylation dependent. Higher methylation levels of L1-EDSBs do not indicate preferential production of methylated L1-EDSBs. When serum-deprived HeLa cells were incubated for 24 hrs with vanillin, sporadic accumulation of hypomethylated L1-EDSBs was observed. This experiment indicated that both methylated and unmethylated L1-EDSBs may be produced. However, unmethylated L1-EDSBs may be immediately repaired.
