# Supplementary material for: Replication independent DNA double-strand break retention may prevent genomic instability
Source: Mol Cancer. 2010 Mar 31;9:70. doi: 10.1186/1476-4598-9-70 (PMC2867818; doi:10.1186/1476-4598-9-70)
Supplement: Additional file 4 — γ-H2AX-bound LINE-1s and radiation. [file 1476-4598-9-70-S4.PDF]

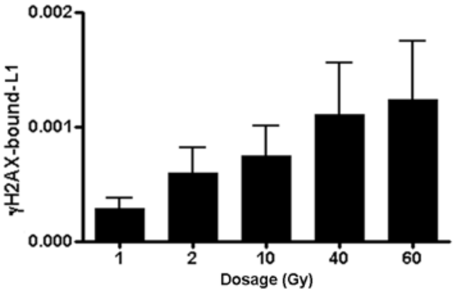

Additional file 4

γ-H2AX-bound LINE-1s and radiation

γ-H2AX-bound LINE-1 genomes per cell in HeLa cells in ice-cold media after exposure to ionizing radiation. An increase in γ-H2AX-bound DNA with increasing doses of radiation was observed. Data represent means ±SEM.
