# Supplementary material for: Replication independent DNA double-strand break retention may prevent genomic instability
Source: Mol Cancer. 2010 Mar 31;9:70. doi: 10.1186/1476-4598-9-70 (PMC2867818; doi:10.1186/1476-4598-9-70)
Supplement: Additional file 5 — γ-H2AX-bound LINE-1s in several cells and cell cycles. [file 1476-4598-9-70-S5.PDF]

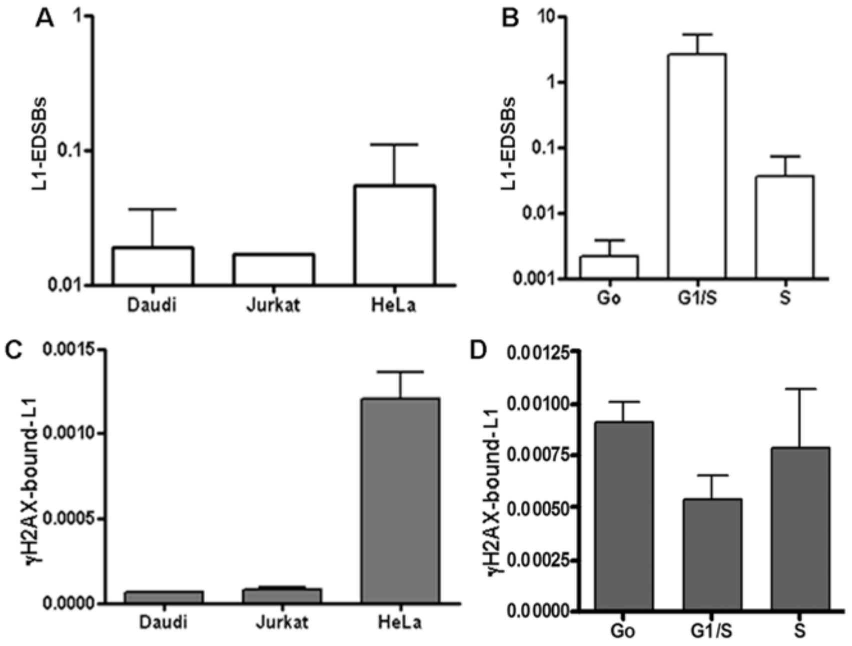

Additional file 5

γ-H2AX-bound LINE-1s in several cells and cell cycles in both non-replicating and replicating phases of the cell cycle, all cells, including leukemic cell lines and HeLa cells, possessed both L1-EDSBs and γ-H2AX-bound LINE-1s. (A, B) L1-EDSB genomes per control genome and (C, D) γ-H2AX-bound LINE-1 genomes per cell. (A, C) Daudi, Jurkat and HeLa cells and (B, D) HeLa cells in the G0, G1/S and S phases. Data represent means ±SEM.
