# Supplementary material for: Replication independent DNA double-strand break retention may prevent genomic instability
Source: Mol Cancer. 2010 Mar 31;9:70. doi: 10.1186/1476-4598-9-70 (PMC2867818; doi:10.1186/1476-4598-9-70)
Supplement: Additional file 6 — L1-RIND-EDSB methylation statuses of Ku86si and Rad51si cells. [file 1476-4598-9-70-S6.PDF]

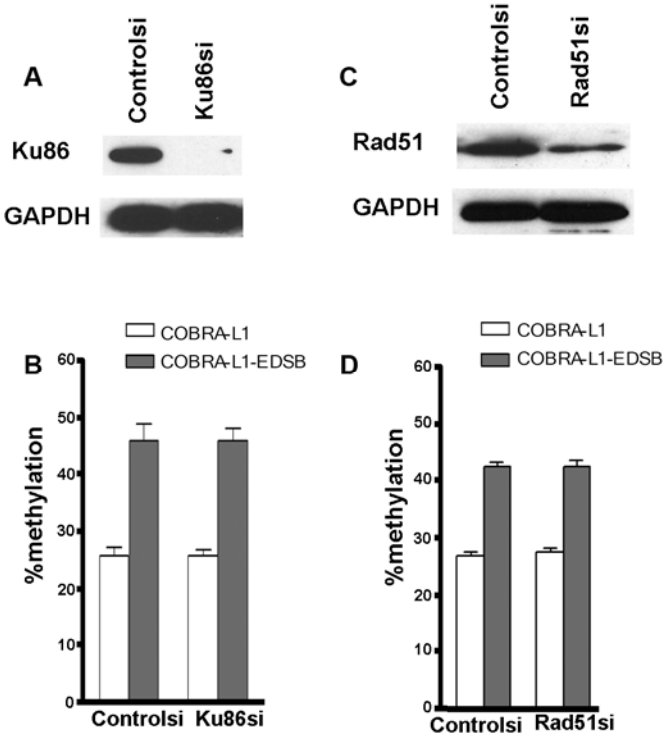

Additional file 6

L1-RIND-EDSB methylation statuses of Ku86si and Rad51si cells

(A) Ku86 and (C) RAD51 levels in control and Ku86 and RAD51 shRNA-transfected HeLa cells. GAPDH is the control. (B and D) COBRA-L1 and COBRA-L1-EDSB analysis of DNA from (B) Ku86 and (D) RAD51 shRNA-transfected HeLa cells. Data represent means ±SEM.
